# Study on Stress Distribution and Its Impact on Reliability of SiO_2_-Based Inorganic Chiplet Gap Filling

**DOI:** 10.3390/mi16121310

**Published:** 2025-11-22

**Authors:** Ziyang Ding, Shaowei Liu, Chen Lin, Tianze Zheng, Lihui Xu, Qiuhan Hu, Tailong Shi, Liyi Li

**Affiliations:** 1 School of Integrated Circuits, Southeast University, Wuxi 214000, China; 220246752@seu.edu.cn (Z.D.); 230240060@seu.edu.cn (S.L.); 220236463@seu.edu.cn (T.Z.); xvlihui@seu.edu.cn (L.X.);; 2 Huawei Technologies Co., Ltd., Shenzhen 518129, China; linchen23@hisilicon.com; 3 School of Advanced Technology, Xi’an Jiaotong-Liverpool University, Suzhou 215123, China; qiuhan.hu23@student.xjtlu.edu.cn

**Keywords:** gap filling, heterogeneous integration, thermomechanical reliability, fracture failure risk

## Abstract

Inorganic gap filling technology is an effective method to improve reliability and heterogeneous integration density in 2.5D and 3D integration. It uses plasma-enhanced chemical vapor deposition (PECVD) to deposit silicon dioxide (SiO_2_) filler layers in gaps between chiplets. This technology is used to replace the Epoxy Mold Compound (EMC) commonly used in traditional packaging. However, as an inorganic filling material, SiO_2_ poses reliability challenges such as cracking and peeling during or after deposition. Furthermore, there lacks quantitative characterization and modeling of the microscale mechanical properties, thermal stress distribution, and fracture failure risk in the filler layer. By combining nanoindentation technology with three-point bending tests, this study reports a comprehensive characterization route for quantitative characterization of mechanical behavior of the filler. A finite element method (FEM) model was also established to predict the thermomechanical reliability of the gap filling process. Raman spectroscopy measured data confirm the model’s reliable predictive ability. The results reveal the impact of filler thickness on the stress. The microscale SiO_2_ mechanical characterization method and the thermal stress and fracture risk FEM prediction model in this study not only address the limitations of traditional testing and simulation but also provide support for process optimization and structural design of gap filling in high-density 2.5D/3D packaging. This work promotes the understanding of inorganic filling process reliability in chiplet integration.

## 1. Introduction

With the rapid development of artificial intelligence, high-performance computing, and Internet of Things technologies, semiconductor devices increasingly demand high-density integration, high-bandwidth interconnection, and low power consumption. Heterogeneous integration (HI) technology, which integrates different functional chiplets in a single package, has become a critical pathway to overcome the bottlenecks of Moore’s Law and achieve system-level performance leaps [1,2,3,4]. In HI architectures, the chiplet gap filling process is a core step to ensure structural stability, electrical performance, and thermal management efficiency. Material selection and process optimization for this step directly impact package reliability and integration density [5,6].

Gap filling technology serves as an effective approach to enhance the density of HI. It employs PECVD to fabricate SiO_2_ filler layers within chiplet gaps. EMC has been commonly used in traditional packaging. EMC typically exhibits a large coefficient of thermal expansion (CTE) mismatch with silicon (Si). It not only causes large warpage but also may lead to chiplet displacement during high-temperature curing [7,8,9,10,11,12]. In contrast, SiO_2_ has gradually become the preferred choice for high-density chiplet gap filling due to its advantages of high thermomechanical compatibility with silicon processes. Specifically, the CTE of SiO_2_ is approximately 0.5 ppm/K, which is closer to that of Si at 2.6 ppm/K [1,2] than that of EMC. For example, Intel’s quasi-monolithic chip (QMC) architecture uses SiO_2_ instead of organic filling materials to achieve near-monolithic interconnect density and thermomechanical stability [1]. Three-dimensional integrated chiplet encapsulation (3D ICE) technology has realized high-density chiplet packaging and vertical interconnection through SiO_2_ reconstruction layers [2].

However, key challenges of inorganic gap filling lie in stress and crack control. Stress distribution is crucial for the reliability of packaging, and studies have shown that stress induced by CTE mismatch directly regulates and leads to failure mechanisms such as crack propagation [13,14,15]. On one hand, residual stress is generated during the deposition of thick-film SiO_2_, and its magnitude is closely related to deposition process parameters (e.g., PECVD power, temperature, and gas ratio) [16,17]. On the other hand, CTE differences between chiplets and filling materials, variations in gap aspect ratios (ARs), and interface interactions in multi-layer stacking lead to complex stress distributions. These factors may cause failures such as wafer warpage, filling layer cracking, or chiplet delamination [5,6,18]. Studies have shown that, when the gap aspect ratio exceeds 0.75, PECVD SiO_2_ tends to form voids due to the “bread loafing” effect, and increasing the aspect ratio further exacerbates stress concentration at corners [19].

This study focuses on the characterization and modeling of the thermal mechanical reliability of gap filling. Mechanical characterization, including nanoindentation and three-point bending tests, were used to systematically characterize the elastic modulus, maximum initial damage stress, fracture toughness, and fracture energy of SiO_2_. A finite element model was established based on the characterized material parameters to analyze thermal stress during gap filling, and Raman spectroscopy was used to validate the model. Additionally, the extended finite element method (XFEM) was employed to analyze the fracture behavior of the model. Comparison of the simulated thermal stress field with the observed locations of cracks induced by thermal and intrinsic stresses was conducted to isolate their respective contributions.

## 2. Experimental

### 2.1. The Preparation of Gap Filling Samples

**Figure 1:**
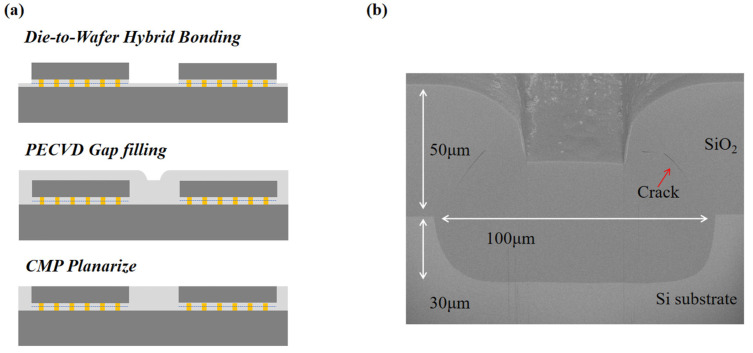
PECVD-fabricated SiO_2_ chiplet gap filling samples: (**a**) schematic of the chiplet gap filling process flow; (**b**) BSE SEM image of the sample cross-section.

This study focuses on multi-chiplet assembly in 2.5D/3D HI processes. The complete process flow is as follows. First, multiple chiplets are precisely secured to the surface of a wafer via die-to-wafer hybrid bonding, forming a chip array with predefined gaps. Subsequently, SiO_2_ is deposited via PECVD to fill these gaps. This step ensures the stability, thermal management capability, and electrical isolation performance of the integrated structure. After the filling process, CMP planarization is used to reconstruct the structure into a wafer-level flat surface, laying the groundwork for subsequent processes such as redistribution layer (RDL) fabrication. The process flow is presented in Figure 1a.

The detailed preparation procedure of the sample is as follows. Chiplet gap structures were fabricated on the 12-inch wafer surface by plasma etching or mechanical processes. The grooves were used for two reasons: first, depending on the bonding quality, the die-to-wafer bonding samples may introduce artifacts, such as delamination at bonding interface due to lack of bonding strength. The artifacts might introduce additional cracks into the gap fill materials, which interfere with the assessment of gap filling step itself. Second, by assuming the bonding interface has sufficient strength, the current samples with grooves are capable of representing the gap filling results by the same geometry. The grooves have a width of 100 μm and a depth of 30 μm. A 50 μm thick SiO_2_ film was deposited on this wafer via PECVD, using tetraethoxysilane (TEOS) as the precursor by Macklin Biochemical Technology Co., Ltd, Shanghai, China. During deposition, the pressure was maintained at 2 torr, with a deposition temperature of 280 °C and a deposition time of about 90 min. The applied power included a high-frequency power of 750 W and a low-frequency power of 210 W. The gas flow rates were set as follows: helium (He) at 4000 sccm, O_2_ at 7000 sccm, and the liquid precursor TEOS was fed at a rate of 3.7 mL/min. The equipment is customized by Southeast University, Nanjing, China. The cross-section of the filled gap under the scanning electron microscope (SEM) in the back-scattered electron (BSE) mode is shown in Figure 1b using a COXEM SEM, Shanghai, China. The image is viewed at a tilting angle of 52°. As can be seen, the gap is completely filled by SiO_2_. Micro-crack can be observed in the SiO_2_ layer at the top corners of the gap.

### 2.2. Nanoindentation Tests

Due to the brittleness of SiO_2_, a major challenge lies in the high fracture risk during the PECVD of SiO_2_ and subsequent grinding processes. This risk is particularly pronounced for chiplet gaps with high aspect ratios [18]. Quantitative assessment of such risk requires accurate mechanical parameters of SiO_2_. But traditional macroscopic mechanical tests (e.g., tensile and bending tests) are limited by sample size and cannot reflect the microscale characteristics of chiplet gap filling layers.

Nanoindentation technology, with its high-precision mechanical characterization capability at the micro/nanoscale, is a suitable solution for SiO_2_ film characterization. Based on the Oliver–Pharr theory [20], this technology can efficiently extract Young’s modulus (*E*) from load–displacement curves, and it has been widely used for characterizing biological, metallic, and semiconductor materials [21,22,23].

A nanoindenter with Berkovich tips was used to measure the mechanical properties of gap filling samples. The load resolution of the instrument is 1 nN, and the displacement resolution is 0.006 nm. The indenter tip is made of diamond. It has a triangular pyramid shape with an inclination angle of 70.3°.

**Figure 2:**
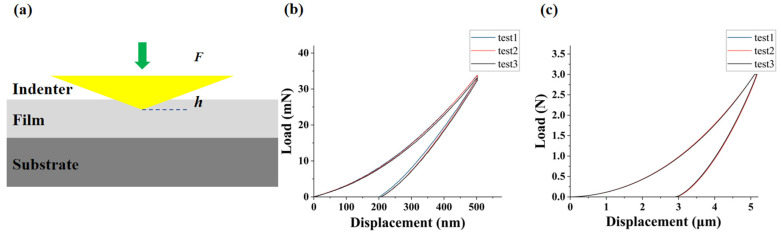
Nanoindentation test principle and load–displacement curve of SiO_2_: (**a**) test schematic; (**b**) nanoindentation load–displacement curve at a 500 nm indentation depth; (**c**) nanoindentation load–displacement curve at a 3N indentation load.

Figure 2a presents the schematic of the nanoindentation test principle. Figure 2b,c depict the load–displacement (P-h) curves for an indentation depth of 500 nm and an indentation load of 3 N, respectively. The curves from multiple experiments exhibit good overlap, which validates the repeatability of the present experimental setup. Specifically, the experimental data for an indentation depth of 500 nm were utilized to determine the elastic modulus, while those for an indentation load of 3 N were utilized to determine the fracture toughness (*K*_c_).

The Young’s modulus can be calculated by:

(1) $$\frac{1}{E_{r}}\;=\;\frac{1-v_{s}^{2}}{E_{s}}\;+\;\frac{1-v_{i}^{2}}{E_{i}},$$

where *E*_i_ is Young’s modulus of indenter, whose value is 1141 GPa; *v*_i_ is Poisson’s ratio of indenter, whose value is 0.07; *E*_s_ is the Young’s modulus of SiO_2_; and *v*_s_ is Poisson’s ratio of SiO_2_, taken as 0.17. *E*_r_ is the reduced modulus, given by Equation (2):

(2) $$E_{r}\;=\;\frac{S}{2\beta }\sqrt{\frac{\pi }{A_{C}}},$$

where *β* is 1.034 for a Berkovich indenter, *A*_C_ is the contact area, and *S* is stiffness, given by Equation (3).

(3) $$S=\frac{dP}{dh},$$

where d*P*/d*h* is the initial slope of the unloading curve.

Fused silica was used as the calibration standard in the experiment, yielding a reduced modulus of 67.77 GPa. The Young’s modulus calculated via Equation (1) was 69.97 GPa, which meets the calibration requirements. The reduced modulus measured for the experimental sample was 58.64 GPa, and the calculated Young’s modulus was 60.02 GPa. This value was adopted as 60 GPa in the subsequent FEM simulations.

*K*_c_ is fracture toughness, which is a key parameter characterizing a material’s ability to resist crack propagation under external stress. As the indenter penetration depth increases, the specimen surface undergoes fracture and spreads outward to the indentation. However, measurable surface cracks cannot be observed at specific corner points of the indentation. Instead, radial cracks formed at its edges. Consequently, calculation methods based on crack length are not applicable here, whereas the energy-based approach is more suitable for calculating fracture toughness.

Based on the loading and unloading curves of nanoindentation, the energy method is used to characterize fracture toughness. According to classical linear elastic fracture mechanics, the energy release rate *G*_c_ under critical conditions is defined as the energy consumed per unit area during crack propagation. Energy release rate is given by Equation (4):

(4) $$G_{c}\;=\;\frac{W_{C}}{A_{\mathrm{fra}}},$$

where *W*_C_ is the fracture energy and *A*_fra_ is the area of the fracture energy release zone (that is, the contact area at the maximum indentation depth). Fracture toughness is defined as the critical stress intensity factor *K*_c_:

(5) $$K_{c}=\sqrt{G_{c}\frac{E}{(1-v^{2})}},$$

The relationship between the total energy (*W*_T_) applied throughout the indentation test and the fracture energy (*W*_c_) can be expressed via energy conservation [24]:

(6) $$W_{C}\;=\;W_{T}-W_{E}-W_{P},$$

where *W*_E_ is elastic energy and *W*_P_ is plastic energy.

Using Equations (4)–(6), the fracture energy (*W*_C_) can be calculated first, and then the fracture toughness can be derived.

### 2.3. Three-Point Bending Test

**Figure 3:**
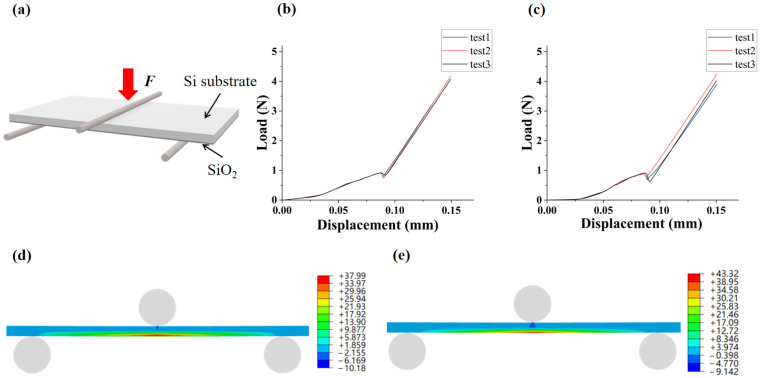
Characterization of SiO_2_ via three-point bending tests: (**a**) test schematic; (**b**) load–displacement curve with the SiO_2_ film facing downward; (**c**) load–displacement curve with the Si substrate facing downward; (**d**) maximum principal stress cloud diagram of the three-point bending testing with the SiO_2_ film facing downward; (**e**) maximum principal stress cloud diagram of the three-point bending testing with the Si substrate facing downward.

The maximum initial damage stress can be defined as the maximum principal stress at the onset of the first crack in the film. Crack initiation can be determined from the abrupt inflection point in the load–displacement curve during the three-point bending test. Figure 3a presents a schematic of the test fixture with the specimen installed. The tester’s sensor exhibits an accuracy of 0.02% of the full-scale range, ensuring the accuracy of the measured results. The film-substrate sample was chosen for three-point bending test. The test samples were machined into rectangular specimens of 40 mm in length and 8 mm in width, and three-point bending tests were conducted. During the tests, the surface of the sample facing downward was subjected to tensile stress.

By reading the load at the abrupt point and inputting it into the finite element model, the corresponding maximum principal stress is obtained. The value derived at this point is the maximum initial damage stress of the material. Since the sample consists of two materials, it is necessary to confirm whether cracks initiate in SiO_2_. To this end, a control experiment was designed. The three-point bending test was performed with the Si substrate facing downward to measure the maximum initial damage stress of Si, thereby verifying whether the parameters measured with the SiO_2_ film facing downward correspond to the maximum initial damage stress of SiO_2_ itself.

Figure 3b,c present the load–displacement curves of the three-point bending tests for configurations where the film faces downward and the substrate faces downward. The average value of the crack initiation load from multiple tests was used as the load input into the finite element method (FEM) model. Figure 3d,e present the contour plots of the maximum principal stress by FEM models for the configurations where the film faces downward and the substrate faces downward under the corresponding loads.

These simulation results provide insights into the stress distribution characteristics under the two configurations. It can be further analyzed to clarify the fracture behavior. When the Si substrate faces downward, the stress concentration point of the maximum principal tensile stress occurs on the Si substrate. Thus, fracture occurs exclusively in the Si substrate. By measuring the maximum principal stress in Si at crack initiation, the maximum initial damage stress of Si is determined to be 43 MPa.

In contrast, when the SiO_2_ film faces downward, the stress concentration point of the maximum principal tensile stress occurs at the interface between the Si substrate and the SiO_2_ film, necessitating the identification of the fracture initiation location. At crack initiation, the maximum principal stresses in SiO_2_ and Si are 21 MPa and 38 MPa, respectively. At this point, the maximum principal stress in the Si substrate is lower than the maximum initial damage stress of Si obtained from the previous experiment. Therefore, crack initiation occurs in the SiO_2_ film, and the maximum principal stress in the SiO_2_ film at this stage corresponds to its maximum initial damage stress.

The mechanical parameters of SiO_2_ characterized in this chapter are listed in Table 1.

### 2.4. Polarized Raman Spectroscopy

In this study, polarized Raman spectroscopy was used to measure the thermomechanical stress of chiplet gap filling structure samples. The principle of Raman spectroscopy for characterizing near-surface stress distribution is as follows: lattice distortion of the Si substrate directly causes Raman spectral shifts. The essence of lattice distortion is crystal structure deformation induced by external forces or internal residual stress. Thus, the local stress state of the material can be deduced from the Raman peak shift.

The correlation coefficient between the Raman shift and stress of the Si surface was calibrated via High-Resolution X-Ray Diffraction (HRXRD) technology. After calibration, they satisfy a linear functional relationship, with the specific expression [25,26]:

(7) σ=−434∆ω,

where *σ* denotes the in-plane biaxial stress and Δ*ω* represents the Raman shift.

**Figure 4:**
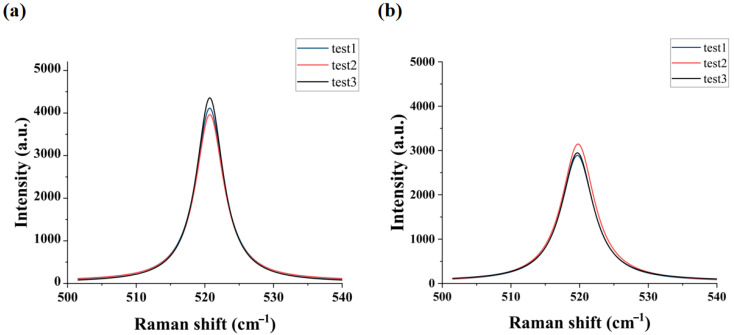
Raman spectral shifts at different measurement temperatures: (**a**) 25 °C; (**b**) 125 °C.

The measurement of Raman frequency was performed with a 532 nm laser. It features a spectral resolution of ±0.02 cm^−1^, corresponding to a stress resolution of approximately 10 MPa. Averaging over multiple measurements was conducted to improve precision. Testing temperatures were set to 25 °C (room temperature) and 125 °C (typical thermal cycling temperature for electronic devices). Multiple characteristic points on the Si substrate were selected for in-plane biaxial stress analysis. The spatial resolution of the Raman spectrometer was approximately 1 μm. Measurements were conducted along the direction parallel to the SiO_2_/Si interface, with the measurement points distributed 5 μm below the SiO_2_/Si interface and within the range of 5 μm to 50 μm from the gap sidewall. The data acquisition interval was 5 μm, resulting in a total of 10 measurement points. Figure 4a,b present the correlation between the Raman shift and intensity of Si at 25 °C and 125 °C, respectively. The average stress values of multiple measurements at each point were used in further analyses.

## 3. Results and Discussion

### 3.1. Validation of the Simulation Was Conducted via Stress Measurement

**Figure 5:**
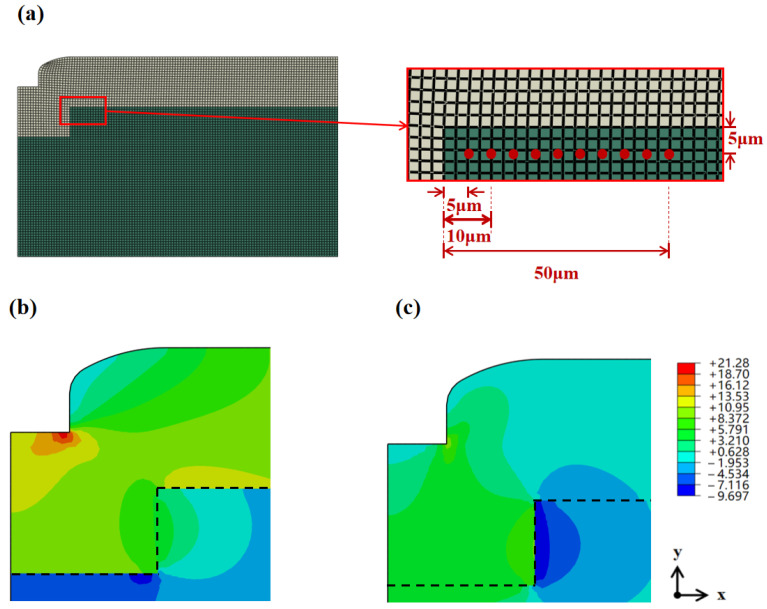
FEM simulations for thermal stress analysis of the gap filling structure: (**a**) geometric structure of the 2D axisymmetric 1/2 model and position of the measurement points in the finite element model; (**b**) lateral stress contour plot of the gap filling structure cross-section at 125 °C; (**c**) longitudinal stress contour plot of the gap filling structure cross-section at 125 °C.

To further analyze thermal stress distribution, a finite element method (FEM) simulation model was established to simulate the gap filling structure in this study. A 2D axisymmetric model was built to analyze the stress under thermal loading. The material parameters used in the model are listed in Table 2. Figure 5a shows the geometric structure and mesh division of the model. This model focuses on thermal stress analysis of structures with a gap depth of 30 μm, width of 100 μm, and 50 μm thick SiO_2_ filler. Considering the localized characteristics of the model, Saint-Venant’s principle was applied for local modeling to simplify calculations while ensuring analysis accuracy of the core region.

Simulated S11 and S22 stress cloud diagrams of the gap filling structure cross-section at 125 °C are presented in Figure 5b,c, respectively. During heating, the CTE of Si is much larger than that of SiO_2_, resulting in a larger free expansion for Si; however, constrained by the SiO_2_ in the gap, Si cannot expand freely and thus bears compressive stress. Correspondingly, SiO_2_ is in a state of tensile stress due to restricting the expansion of Si.

**Figure 6:**
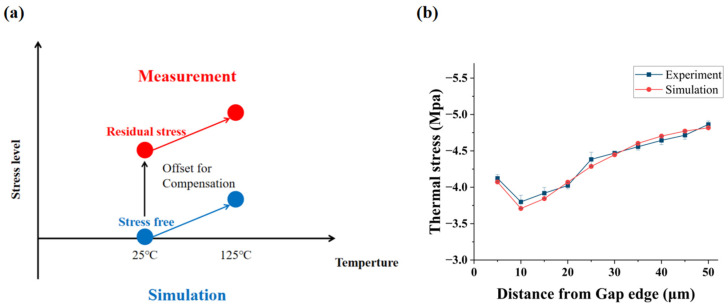
Comparison between FEM simulation and Raman measurement data for thermal stress of gap filling structure samples: (**a**) stress compensation scheme. Using the residual stress measured at room temperature (25 °C) as a compensation offset to eliminate the deviation caused by the “simulation assumption of zero stress at room temperature”, enabling stress data matching between 25 °C and 125 °C. (**b**) Comparison of in-plane biaxial stress between simulation and measurement data.

To verify the accuracy of the simulation model, the thermal stress simulation results were compared with experimental measurement data. The longitudinal and lateral thermal stresses at corresponding positions were extracted from the simulation to achieve precise matching with the measurement data. Simultaneously, the residual stress measured at room temperature (25 °C) was used as a compensation offset to eliminate the deviation caused by the simulation assumption of zero stress at room temperature (Figure 6a). As shown in Figure 6b, the simulation results and experimental results show good agreement and exhibit consistent variation trends.

Under heating conditions, the CTE of the Si substrate is significantly higher than that of the SiO_2_ filling layer. In the region close to the gap, the dominant localized constraint along the transverse direction results in a high initial value of S11 and a rapid rate of decrease. In contrast, the localized constraint along the longitudinal direction is weaker, leading to a low initial value of S22 and a slow rate of increase. Meanwhile, the decreasing rate of S11 exceeding the increasing rate of S22.

As the distance from the gap increases, the constraint on the Si substrate transitions from localized concentration to global deformation. The decreasing rate of S11 diminishes as the localized constraint is gradually released, while the increasing rate of S22 accelerates and eventually surpasses that of S11 as the substrate’s thermal expansion demand shifts toward the longitudinal direction. Consequently, the in-plane biaxial stress first decreases and then increases with increasing distance from the gap.

### 3.2. Simulation of SiO_2_ Crack Initiation and Propagation via the XFEM

To gain in-depth insight into the fracture behavior of SiO_2_ in the gap filling structure under process-induced thermal loading, this study used the XFEM to numerically simulate the initiation and propagation of cracks in the SiO_2_ layer. The XFEM does not require predefining crack paths and allows cracks to propagate along arbitrary paths, making it suitable for simulating the fracture behavior of brittle materials under complex stress states.

The physical process focused on in this simulation is as follows: after PECVD, when the sample cools from the deposition high temperature (280 °C) to room temperature, thermally mismatched stress is generated due to CTE differences between materials, which, in turn, induces fracture.

Based on the geometric structure and material parameters of the aforementioned FEM model (Table 2), the XFEM module was enabled to simulate the fracture of the SiO_2_ layer in the gap filling structure. The fracture parameters of SiO_2_ in the model used the values measured via nanoindentation and three-point bending tests (Table 1). The simulation assumed an initial crack-free structure. Moreover, the maximum principal stress criterion was used to determine crack initiation under thermal loading between 280 °C and 25 °C. When the maximum principal stress at a point in SiO_2_ exceeds maximum initial damage stress, that point is identified as a crack initiation site. Subsequently, the energy release rate criterion was used to simulate the crack propagation path.

**Figure 7:**
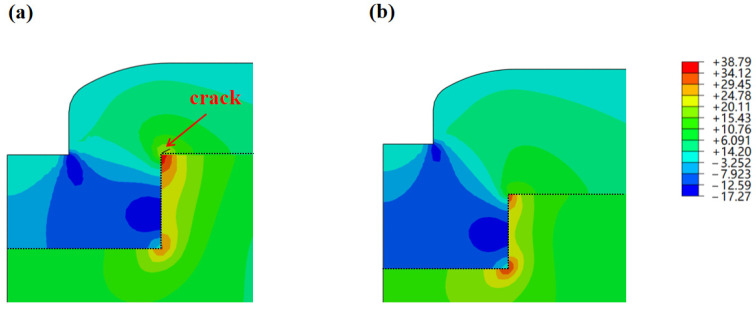
Contour plots of maximum principal stress from XFEM simulation: (**a**) 30 μm thick filling layer; (**b**) 50 μm thick filling layer.

Figure 7a,b present the maximum principal stress distributions in the models featuring a 100 μm wide and 50 μm deep gap, with 30 μm thick and 50 μm thick filling layers deposited inside, respectively, after cooling to room temperature. The CTE of SiO_2_ is much lower than that of Si. During cooling, the Si substrate shrinks much more significantly than the SiO_2_ filling layer does. This results in deformation constraints at the interface between the two materials. At this point, the in-plane normal stresses in different directions generated by the shrinkage mismatch cannot be freely released and thus convert into shear stress at the interface. This leads to stress concentration of S12 at the upper corner of the gap, which, in turn, makes this location a stress concentration point for the maximum principal tensile stress. Simulation results indicate that, for the model with a 30 μm thick filling layer, the maximum principal stress at the upper corner of the gap edge first exceeds the critical value and, therefore, cracks are initiated at this location. However, when the filling layer thickness is 50 μm, the maximum principal stress of the model is lower than that of the model with a 30 μm thick filling layer. Meanwhile, the maximum principal stress in the 50 μm thick filling layer is also lower than the material maximum initial damage stress, so no cracks are generated. This is because a greater amount of SiO_2_ filling layer in the gap results in greater compression, which causes S11 (compressive stress component) to exhibit a larger magnitude while reducing the magnitude of S12. This leads to a decrease in the maximum principal stress of the model with a 50 μm thick filling layer. This indicates that the model with the 50 μm thick filling layer exhibits higher thermomechanical reliability relative to the one with the 30 μm thick filling layer.

Residual stress inside the sample is typically composed of thermal stress and intrinsic stress [27]. In this section, the FEM is used to analyze the thermal stress of the model and the corresponding fracture behavior. In the FEM model, the sample with a 50 μm thick filling layer did not fracture. However, in actual samples, cracks appeared inside the filling layer (Figure 1b). The location where the cracks appeared was not a region of thermal stress concentration. This indicates the significant role of intrinsic stress in crack initiation and propagation.

## 4. Conclusions

This study addresses the reliability challenges associated with SiO_2_-based inorganic chiplet gap filling in HI, including the difficulty of the microscale mechanical characterization, unclear thermal stress distribution, and ambiguous fracture risk.

Using nanoindentation and three-point bending tests, the mechanical properties of SiO_2_ were quantified, including Young’s modulus, maximum initial damage stress, fracture toughness, and fracture energy. Specifically, for our PECVD SiO_2_ material, Young’s modulus is 60 GPa, maximum initial damage stress is 21 MPa, fracture toughness is 0.914 MPa·m^1/2^, and fracture energy is 13.5 J/m^2^.

Polarized Raman spectroscopy was used to measure stresses at 25 °C and 125 °C, revealing compressive stress in Si and tensile stress in SiO_2_, which arose from CTE mismatch. A 2D axisymmetric FEM model, calibrated with room-temperature residual stress, exhibits good agreement with the Raman-derived stress data.

XFEM simulations cooling from 280 °C to 25 °C indicate that 30 μm thick SiO_2_ films will crack at the upper corners of the gap, whereas 50 μm thick SiO_2_ films remain intact, highlighting the critical role of filling layer thickness in mitigating the fracture risks and intrinsic stress’s significant role in crack initiation and propagation.

This work addresses gaps in traditional testing and simulation approaches, providing a framework for reliability assessment and supporting the optimization of 2.5D/3D packaging, advancing the application of SiO_2_ gap filling in HI.

## Figures and Tables

**Table 1:** Mechanical parameters of SiO_2_.

Young’s Modulus (GPa)	Maximum Initial Damage Stress (MPa)	Fracture Toughness (MPa·m^1/2^)	Fracture Energy (J/m^2^)
60	21	0.914	13.5

**Table 2:** Material parameters for FEM simulation.

Material	Young’s Modulus (GPa)	Poisson’s Ratio
Si	130	0.26
SiO_2_	60	0.17

## Data Availability

The original contributions presented in this study are included in the article. Further inquiries can be directed to the corresponding author.
